# Active inference and learning

**DOI:** 10.1016/j.neubiorev.2016.06.022

**Published:** 2016-09

**Authors:** Karl Friston, Thomas FitzGerald, Francesco Rigoli, Philipp Schwartenbeck, John O’Doherty, Giovanni Pezzulo

**Affiliations:** a The Wellcome Trust Centre for Neuroimaging, UCL, 12 Queen Square, London, United Kingdom; b Max-Planck—UCL Centre for Computational Psychiatry and Ageing Research, London, United Kingdom; c Centre for Neurocognitive Research, University of Salzburg, Salzburg, Austria; d Neuroscience Institute, Christian-Doppler-Klinik, Paracelsus Medical University Salzburg, Salzburg, Austria; e Caltech Brain Imaging Center, California Institute of Technology, Pasadena, USA; f Institute of Cognitive Sciences and Technologies, National Research Council, Rome, Italy

**Keywords:** Active inference, Habit learning, Bayesian inference, Goal-directed, Free energy, Information gain, Bayesian surprise, Epistemic value, Exploration, Exploitation

## Abstract

This paper offers an active inference account of choice behaviour and learning. It focuses on the distinction between goal-directed and habitual behaviour and how they contextualise each other. We show that habits emerge naturally (and autodidactically) from *sequential policy* optimisation when agents are equipped with *state-action policies*. In active inference, behaviour has explorative (epistemic) and exploitative (pragmatic) aspects that are sensitive to ambiguity and risk respectively, where epistemic (ambiguity-resolving) behaviour enables pragmatic (reward-seeking) behaviour and the subsequent emergence of habits. Although goal-directed and habitual policies are usually associated with *model-based* and *model-free* schemes, we find the more important distinction is between *belief-free* and *belief-based* schemes. The underlying (variational) belief updating provides a comprehensive (if metaphorical) process theory for several phenomena, including the transfer of dopamine responses, reversal learning, habit formation and devaluation. Finally, we show that active inference reduces to a classical (Bellman) scheme, in the absence of ambiguity.

## Highlights

• Optimal behaviour is quintessentially belief based.
• Behaviour can be described as optimising expected free energy.
• Expected free energy entails pragmatic and epistemic value.
• Habits are learned by observing one’s own goal directed behaviour.
• Habits are then selected online during active inference.

## 1 Introduction

There are many perspectives on the distinction between goal-directed and habitual behaviour (Balleine and Dickinson, 1998, Yin and Knowlton, 2006, Keramati et al., 2011, Dezfouli and Balleine, 2013, Dolan and Dayan, 2013, Pezzulo et al., 2013). One popular view rests upon model-based and model-free learning (Daw et al., 2005, Daw et al., 2011). In model-free approaches, the value of a state (e.g., being in a particular location) is learned through trial and error, while actions are chosen to maximise the value of the next state (e.g. being at a rewarded location). In contrast, model-based schemes compute a value-function of states under a model of behavioural contingencies (Gläscher et al., 2010). In this paper, we consider a related distinction; namely, the distinction between policies that rest upon beliefs about states and those that do not. In other words, we consider the distinction between choices that depend upon a (free energy) functional of beliefs about states, as opposed to a (value) function of states.

Selecting actions based upon the value of states only works when the states are known. In other words, a value function is only useful if there is no ambiguity about the states to which the value function is applied. Here, we consider the more general problem of behaving under ambiguity (Pearson et al., 2014). Ambiguity is characterized by an uncertain mapping between hidden states and outcomes (e.g., states that are partially observed) – and generally calls for policy selection or decisions under uncertainty; e.g. (Alagoz et al., 2010, Ravindran, 2013). In this setting, optimal behaviour depends upon beliefs about states, as opposed to states *per se*. This means that choices necessarily rest on inference, where optimal choices must first resolve ambiguity. We will see that this resolution, through epistemic behaviour, is an emergent property of (active) inference under prior preferences or goals. These preferences are simply outcomes that an agent or phenotype expects to encounter (Friston et al., 2015). So, can habits be learned in an ambiguous world? In this paper, we show that epistemic habits emerge naturally from observing the consequences of (one’s own) goal-directed behaviour. This follows from the fact that ambiguity can be resolved, unambiguously, by epistemic actions.

To illustrate the distinction between belief-based and belief-free policies, consider the following examples: a predator (e.g., an owl) has to locate a prey (e.g., a field mouse). In this instance, the best goal-directed behaviour would be to move to a vantage point (e.g., overhead) to resolve ambiguity about the prey’s location. The corresponding belief-free policy would be to fly straight to the prey, from any position, and consume it. Clearly, this belief-free approach will only work if the prey reveals its location unambiguously (and the owl knows exactly where it is). A similar example could be a predator waiting for the return of its prey to a waterhole. In this instance, the choice of whether to wait depends on the time elapsed since the prey last watered. The common aspect of these examples is that the belief state of the agent determines the optimal behaviour. In the first example, this involves soliciting cues from the environment that resolve ambiguity about the context (e.g., location of a prey). In the second, optimal behaviour depends upon beliefs about the past (i.e., memory). In both instances, a value-function of the states of the world cannot specify behaviour, because behaviour depends on beliefs or knowledge (i.e., *belief states* as opposed to states of the world).

Usually, in Markov decision processes (MDP), belief-based problems call for an augmented state-space that covers the belief or information states of an agent (Averbeck, 2015) – known as a belief MDP (Oliehoek et al., 2005). Although this is an elegant solution to optimising policies under uncertainty about (partially observed) states, the composition of belief states can become computationally intractable; not least because belief MDPs are defined over a continuous belief state-space (Cooper, 1988, Duff, 2002, Bonet and Geffner, 2014). Active inference offers a simpler approach by absorbing any value-function into a single functional of beliefs. This functional is variational free energy that scores the surprise or uncertainty associated with a belief, in light of observed (or expected) outcomes. This means that acting to minimise free energy resolves ambiguity and realises unsurprising or preferred outcomes. We will see that this single objective function can be unpacked in a number of ways that fit comfortably with established formulations of optimal choice behaviour and foraging.

In summary, schemes that optimise state-action mappings – via a value-function of states – could be considered as habitual, whereas goal-directed behaviour is quintessentially belief-based. This begs the question as to whether habits can emerge under belief-based schemes like active inference. In other words, can habits be learned by simply observing one’s own goal-directed behaviour? We show this is the case; moreover, habit formation is an inevitable consequence of equipping agents with the hypothesis that habits are sufficient to attain goals. We illustrate these points, using formal (information theoretic) arguments and simulations. These simulations are based upon a generic (variational) belief update scheme that shows several behaviours reminiscent of real neuronal and behavioural responses. We highlight some of these behaviours in an effort to establish the construct validity of active inference.

This paper comprises four sections. The first provides a description of active inference, which combines our earlier formulations of planning as inference (Friston et al., 2014) with Bayesian model averaging (FitzGerald et al., 2014) and learning (FitzGerald et al., 2015a, FitzGerald et al., 2015b). Importantly, action (i.e. policy selection), perception (i.e., state estimation) and learning (i.e., reinforcement learning) all minimise the same quantity; namely, variational free energy. In this formulation, habits are learned under the assumption (or hypothesis) there is an optimal mapping from one state to the next, that is not context or time-sensitive.^1^ Our key interest was to see if habit-learning emerges as a Bayes-optimal *habitisation* of goal-directed behaviour, when circumstances permit. This follows a general line of thinking, where habits are effectively learned as the invariant aspects of goal-directed behaviour (Dezfouli and Balleine, 2013, Pezzulo et al., 2013, Pezzulo et al., 2014, Pezzulo et al., 2015). It also speaks to the *arbitration* between goal-directed and habitual policies (Lee et al., 2014). The second section considers variational belief updating from the perspective of standard approaches to policy optimisation based on the Bellman optimality principle. In brief, we will look at dynamic programming schemes for Markovian decision processes that are cast in terms of value-functions – and how the ensuing value (or policy) iteration schemes can be understood in terms of active inference.

The third section uses simulations of foraging in a radial maze to illustrate some key aspects of inference and learning; such as the transfer of dopamine responses to conditioned stimuli, as agents become familiar with their environmental contingencies (Fiorillo et al., 2003). The final section considers context and habit learning, concluding with simulations of reversal learning, habit formation and devaluation (Balleine and Ostlund, 2007). The aim of these simulations is to illustrate how the above phenomena emerge from a single imperative (to minimise free energy) and how they follow naturally from each other.

## 2 Active inference and learning

This section provides a brief overview of active inference. The formalism used in this paper builds upon our previous treatments of Markov decision processes (Schwartenbeck et al., 2013, Friston et al., 2014, Friston et al., 2015, Pezzulo et al., 2015, Pezzulo et al., 2016). Specifically, we extend sequential policy optimisation to include action-state policies of the sort optimised by dynamic programming and backwards induction (Bellman, 1952, Howard, 1960). Active inference is based upon the premise that everything minimises variational free energy. This leads to some surprisingly simple update rules for action, perception, policy selection, learning and the encoding of uncertainty (i.e., precision) that generalise established normative approaches.

In principle, the following scheme can be applied to any paradigm or choice behaviour. Earlier applications have been used to model waiting games (Friston et al., 2013) the urn task and evidence accumulation (FitzGerald et al., 2015a, FitzGerald et al., 2015b), trust games from behavioural economics (Moutoussis et al., 2014, Schwartenbeck et al., 2015a, Schwartenbeck et al., 2015b), addictive behaviour (Schwartenbeck et al., 2015c), two-step maze tasks (Friston et al., 2015) and engineering benchmarks such as the mountain car problem (Friston et al., 2012a). Empirically, it is has been used in the setting of computational fMRI (Schwartenbeck et al., 2015a). More generally, in theoretical biology, active inference is a necessary aspect of any biological self-organisation (Friston, 2013), where free energy reflects survival probability in an evolutionary setting (Sella and Hirsh, 2005).

In brief, active inference separates the problems of optimising action and perception by assuming that action fulfils predictions based upon perceptual inference or state-estimation. Optimal predictions are based on (sensory) evidence that is evaluated in relation to a *generative model* of (observed) outcomes. This allows one to frame behaviour as fulfilling optimistic predictions, where the inherent optimism is prescribed by prior preferences (Friston et al., 2014). Crucially, the generative model contains beliefs about future states and policies, where the most likely policies lead to preferred outcomes. This enables action to realise preferred outcomes, based on the assumption that both action and perception are trying to maximise the evidence or marginal likelihood of the generative model, as scored by variational free energy.

**Fig. 1:**
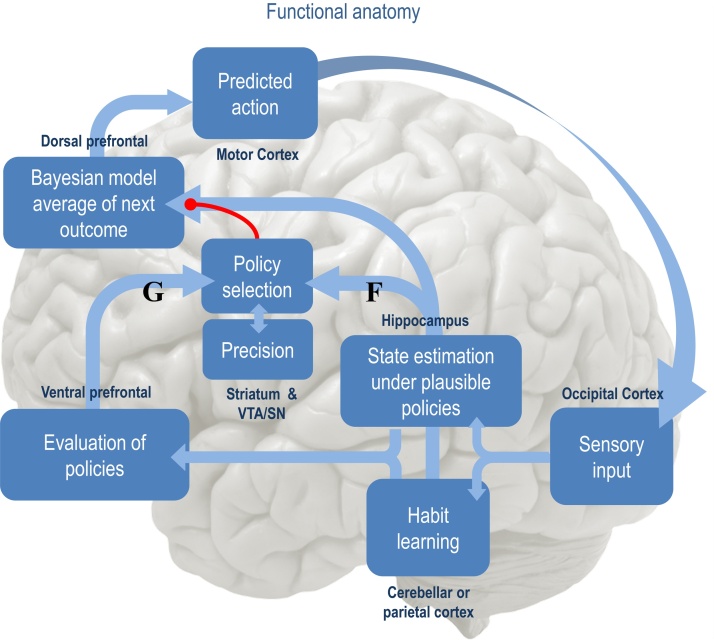
The functional anatomy of belief updating: sensory evidence is accumulated to optimise expectations about the current state, which are constrained by expectations of past (and future) states. This corresponds to state estimation under each policy the agent entertainments. The quality of each policy is evaluated in the ventral prefrontal cortex – possibly in combination with ventral striatum (van der Meer et al., 2012) – in terms of its expected free energy. This evaluation and the ensuing policy selection rest on expectations about future states. Note that the explicit encoding of future states lends this scheme the ability to plan and explore. After the free energy of each policy has been evaluated, it is used to predict the subsequent hidden state through Bayesian model averaging (over policies). This enables an action to be selected that is most likely to realise the predicted state. Once an action has been selected, it generates a new observation and the cycle begins again. Fig. 2 illustrates the formal basis of this computational anatomy, in terms of belief updating.

Fig. 1, provides an overview of active inference in terms of the functional anatomy and processes implicit in the minimisation of variational free energy. In brief, sensory evidence is accumulated to form beliefs about the current state of the world. These beliefs are constrained by expectations of past (and future) states. This evidence accumulation corresponds to state estimation under each policy the agent entertainments. The quality of each policy is then evaluated in terms of its expected free energy. The implicit policy selection therefore depends on expectations about future states under each policy, where the encoding of future states lends the scheme an ability to plan and explore. After the free energies of each policy have been evaluated, they are used to predict the next state of the world, through Bayesian model averaging (over policies); in other words, policies that lead to preferred outcomes have a greater influence on predictions. This enables action to realise predicted states. Once an action has been selected, it generates a new observation and the perception-action cycle begins again. In what follows, we will see how these processes emerge naturally from the single imperative to minimise (expected) free energy, under a fairly generic model of the world.

As noted above, the generative model includes hidden states in the past and the future. This enables agents to select policies that will maximise model evidence in the future by minimising expected free energy. Furthermore, it enables learning about contingencies based upon state transitions that are inferred retrospectively. We will see that this leads to a Bayes-optimal arbitration between epistemic (explorative) and pragmatic (exploitative) behaviour that is formally related to several established constructs; e.g., the Infomax principle (Linsker, 1990), Bayesian surprise (Itti and Baldi, 2009), the value of information (Howard, 1966), artificial curiosity (Schmidhuber, 1991), expected utility theory (Zak, 2004) and so on. We start by describing the generative model upon which predictions and actions are based. We then describe how action is specified by (Bayesian model averages of) beliefs about states of the world, under different models or policies. This section concludes by considering the optimisation of these beliefs (i.e., inference and learning) through Bayesian belief updating. The third section illustrates the formalism of the current section, using an intuitive example.

Notation The parameters of categorical distributions over discrete states s∈{0,1} are denoted by column vectors of expectations s∈{0,1}, while the ∼ notation denotes sequences of variables over time; e.g., $\tilde{s}=(s_{1},\ldots ,s_{T})$. The entropy of a probability distribution P(s)=Pr(S=s) is denoted by $H(S)=H[P(s)]=E_{P}[-\ln P(s)]$, while the relative entropy or Kullback-Leibler (KL) divergence is denoted by $D[Q(s)||P(s)]=E_{Q}[\ln Q(s)-\ln P(s)]$. Inner and outer products are indicated by $A\cdot B=A^{T}B$, and $A⊗B=AB^{T}$ respectively. We use a hat notation  to denote (natural) logarithms. Finally, P(o|s)=Cat(A) implies $\mathrm{Pr}(o=i|s=j)=Cat(A_{ij})$.

Definition Active inference rests on the tuple (O,P,Q,R,S,T,U):

• A finite set of outcomes *O*
• A finite set of control states or actions *U*
• A finite set of hidden states *S*
• A finite set of time sensitive policies *T*
• A *generative process* $R(\tilde{o},\tilde{s},\tilde{u})$ that generates probabilistic outcomes o∈O from (hidden) states s∈S and action u∈U
• A *generative model* $P(\tilde{o},\tilde{s},\pi ,\eta )$ with parameters η, over outcomes, states and policies π∈T, where π∈{0,…,K} returns a sequence of actions $u_{t}=\pi (t)$
• An *approximate posterior* $Q(\tilde{s},\pi ,\eta )=Q(s_{0}|\pi )\ldots Q(s_{T}|\pi )Q(\pi )Q(\eta )$ over states, policies and parameters with expectations $\left(s_{0}^{\pi },\ldots ,s_{T}^{\pi },\pi ,\eta \right)$

Remarks The *generative process* describes transitions among (hidden) states in the world that generate observed outcomes. These transitions depend upon actions, which depend on beliefs about the next state. In turn, these beliefs are formed using a *generative model* of how observations are generated. The generative model describes what the agent believes about the world, where beliefs about hidden states and policies are encoded by expectations. Note the distinction between actions (that are part of the generative process in the world) and policies (that are part of the generative model of an agent). This distinction allows actions to be specified by beliefs about policies, effectively converting an optimal control problem into an optimal inference problem (Attias, 2003, Botvinick and Toussaint, 2012).

### 2.1 The generative model

The generative model for partially observable Markov decision processes can be parameterised in a general way as follows, where the model parameters are η={a,b,c,d,e,β}:
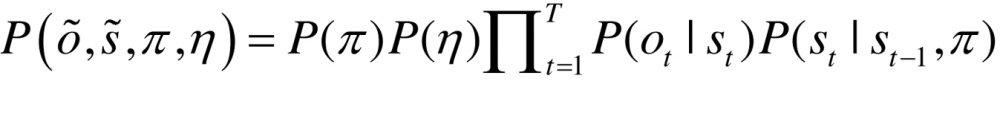
(1.a)
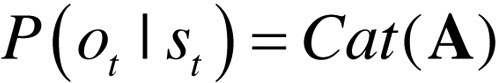
(1.b)
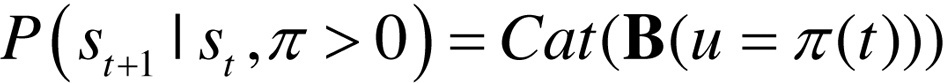
(1.c)
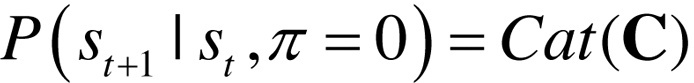
(1.d)
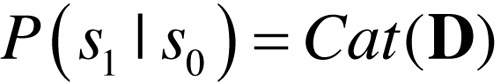
(1.e)
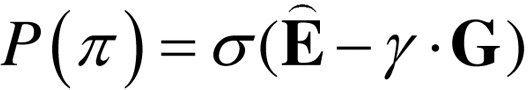

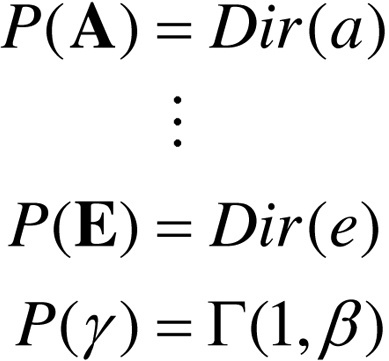


The role of each model parameter will be unpacked when we consider model inversion and worked examples. For reference, Table 1 provides a brief description of this model’s states and parameters. The corresponding (approximate) posterior over hidden states and parameters $x=(\tilde{s},\pi ,\eta )$ can be expressed in terms of their expectations $x=(s_{0}^{\pi },\ldots ,s_{T}^{\pi },\pi ,\eta )$ and η=(a,b,c,d,e,β)(2)
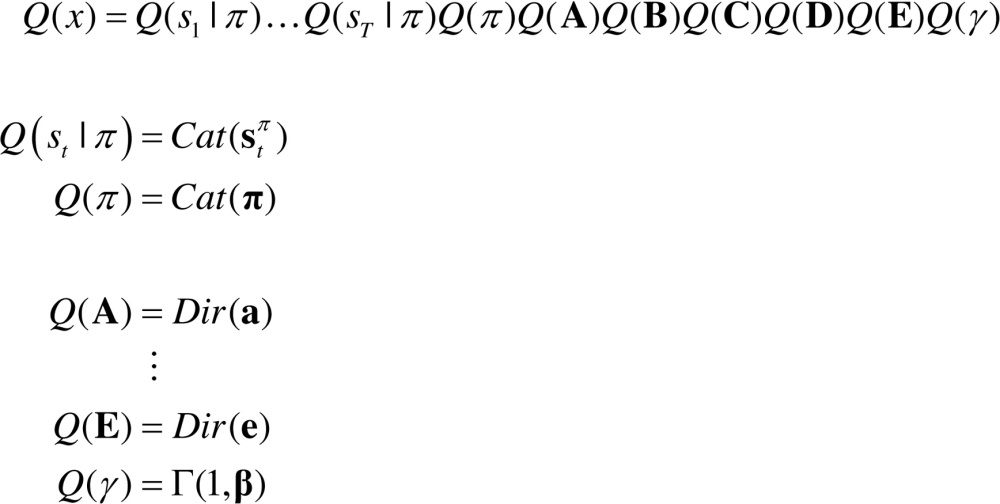


In this generative model, observations depend only upon the current state (Eq. (1.a)), while state transitions depend on a policy or sequence of actions (Eq. (1.b)). This (sequential) policy is sampled from a Gibbs distribution or softmax function of expected free energy , with inverse temperature or precision γ (Eq. (1.e)). Here **E** corresponds to prior beliefs about policies, while **G** is the free energy expected under each policy (see below). Crucially, policies come in two flavours: when π=0 the state transitions do not depend on the policy and the next state is always specified (probabilistically) by the current state (Eq. (1.c)). In other words, there is one special policy that, if selected, will generate the same state transitions and subsequent actions, irrespective of time or context. This is the *habitual* or state-action policy. Conversely, when π>0, transitions depend on a sequential policy that entails ordered sequences of actions (Eq. (1.b)).

Note that the policy is a random variable that has to be inferred. In other words, the agent entertains competing hypotheses or models of its behaviour, in terms of policies. This contrasts with standard formulations, in which one (habitual) policy returns an action as a function of each state u=π(s), as opposed to time, u=π(t). In other words, different policies can prescribe different actions from the same state, which is not possible under a state-action policy. Note also that the approximate posterior is parameterised in terms of expected states under each policy. In other words, we assume that the agent keeps a separate record of expected states – in the past and future – for each allowable policy. Essentially, this assumes the agents have a short term memory for prediction and postdiction. When interpreted in the light of hippocampal dynamics, this provides a simple explanation for phenomena like place-cell responses and phase precession (Friston and Buzsaki, 2016). A separate representation of trajectories for each policy can be thought of in terms of a saliency map, where each location corresponds to a putative policy: e.g., a fixation point for the next saccade (Friston et al., 2012b, Mirza et al., 2016).

The predictions that guide action are based upon a *Bayesian model average* of policy-specific states. In other words, policies the agent considers it is more likely to be pursuing dominate predictions about the next outcome and the ensuing action. Finally, all the conditional probabilities – including the initial state – are parameterised in terms of Dirichlet distributions (FitzGerald et al., 2015b). The sufficient statistics of these distributions are concentration parameters that can be regarded as the number of [co]occurrences encountered in the past. In other words, they encode the number of times various combinations of states and outcomes have been observed, which specify their probability – and the confidence in that probability. In what follows, we first describe how actions are selected, given beliefs about the hidden state of the world and the policies currently being pursued. We will then turn to the more difficult problem of optimising the beliefs upon which action is based.

### 2.2 Behaviour action and reflexes

We associate action with reflexes that minimise the expected KL divergence between the outcomes predicted at the next time step and the outcome predicted after each action. Mathematically, this can be expressed in terms of minimising (outcome) prediction errors as follows:

 (3)

This formulation of action is considered reflexive by analogy to motor reflexes that minimise the discrepancy between proprioceptive signals (primary afferents) and descending motor commands or predictions. Heuristically, action realises expected outcomes by minimising the expected outcome prediction error. Expectations about the next outcome therefore enslave behaviour. If we regard competing policies as models of behaviour, the predicted outcome is formally equivalent to a Bayesian model average of outcomes, under posterior beliefs about policies (last equality above).

### 2.3 Free energy and expected free energy

In active inference, all the heavy lifting is done by minimising free energy with respect to expectations about hidden states, policies and parameters. Variational free energy can be expressed as a function of the approximate posterior in a number of ways:(4)
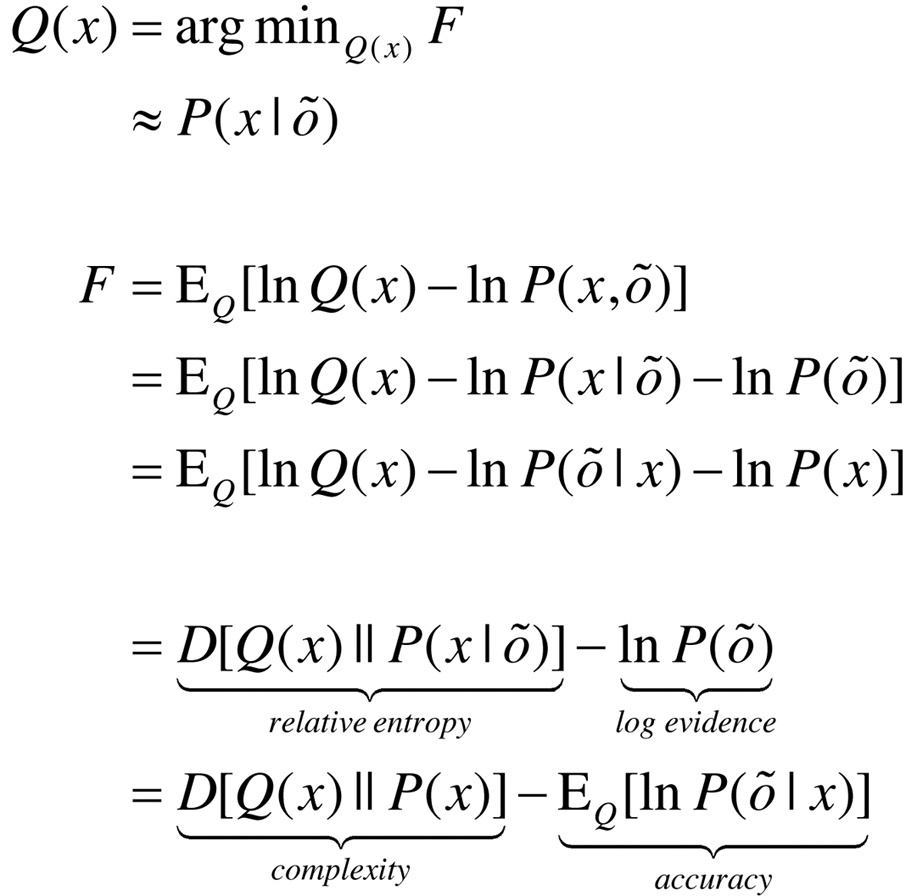
where $\tilde{o}=(o_{1},\ldots ,o_{t})$ denotes observations up until the current time.

Because KL divergences cannot be less than zero, the penultimate equality means that free energy is minimised when the approximate posterior becomes the true posterior. At this point, the free energy becomes the negative log evidence for the generative model (Beal, 2003). This means minimising free energy is equivalent to maximising model evidence, which is equivalent to minimising the complexity of accurate explanations for observed outcomes (last equality).

With this equivalence in mind, we now turn to the prior beliefs about policies that shape posterior beliefs − and the Bayesian model averaging that determines action. Minimising free energy with respect to expectations ensures that they encode posterior beliefs, given observed outcomes. However, beliefs about policies rest on outcomes in the future, because these beliefs determine action and action determines subsequent outcomes. This means that policies should, a priori, minimise the free energy of beliefs about the future. Eq. (1.e) expresses this formally by making the log probability of a policy proportional to the free energy expected under that policy. The expected free energy of a policy follows from Eq. (4) (Friston et al., 2015).(5)
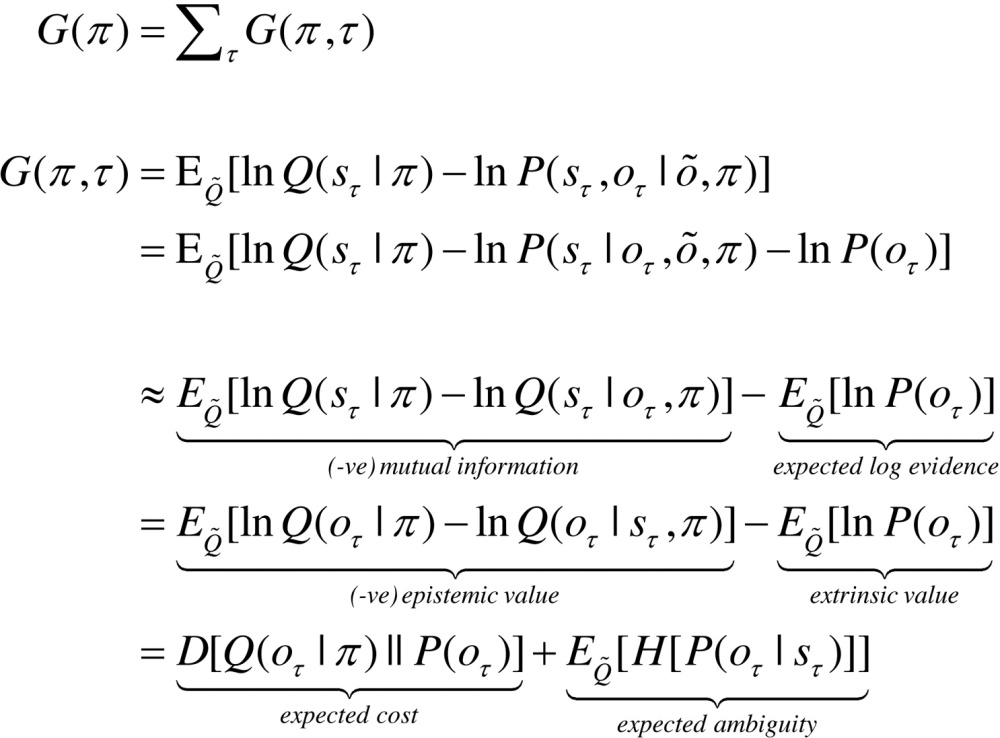
where $\tilde{Q}=Q(o_{\tau },s_{\tau }|\pi )=P(o_{\tau }|s_{\tau })Q(s_{\tau }|\pi )\approx P(o_{\tau },s_{\tau }|\tilde{o},\pi )$ and $Q(o_{\tau }|s_{\tau },\pi )=P(o_{\tau }|s_{\tau })$.

In the expected free energy, relative entropy becomes mutual information and log-evidence becomes the log-evidence expected under the predicted outcomes. If we associate the log prior over outcomes with utility or prior preferences: $U(o_{\tau })=\ln P(o_{\tau })$, the expected free energy can also be expressed in terms of epistemic and extrinsic value. This means extrinsic value corresponds to expected utility and can be associated with the log-evidence for an agent’s model of the world expected in the future. Epistemic value is simply the expected information gain (mutual information) afforded to hidden states by future outcomes (or vice-versa). A final re-arrangement shows that complexity becomes expected cost; namely, the KL divergence between the posterior predictions and prior preferences; while accuracy becomes the accuracy, expected under predicted outcomes (i.e. negative ambiguity). This last equality shows how expected free energy can be evaluated relatively easily: it is just the divergence between the predicted and preferred outcomes, minus the ambiguity (i.e., entropy) expected under predicted states.

In summary, expected free energy is defined in relation to prior beliefs about future outcomes. These define the expected cost or complexity and complete the generative model. It is these preferences that lend inference and action a purposeful or pragmatic (goal directed) aspect. There are several useful interpretations of expected free energy that appeal to (and contextualise) established constructs. For example, maximising epistemic value is equivalent to maximising (expected) Bayesian surprise (Schmidhuber, 1991, Itti and Baldi, 2009), where Bayesian surprise is the KL divergence between posterior and prior beliefs. This can also be interpreted in terms of the principle of maximum mutual information or minimum redundancy (Barlow, 1961, Linsker, 1990, Olshausen and Field, 1996, Laughlin, 2001). This is because epistemic value is the mutual information between hidden states and observations. In other words, it reports the reduction in uncertainty about hidden states afforded by observations. Because the KL divergence (or information gain) cannot be less than zero, it disappears when the (predictive) posterior is not informed by new observations. Heuristically, this means epistemic policies will search out observations that resolve uncertainty about the state of the world (e.g., foraging to locate a prey). However, when there is no posterior uncertainty – and the agent is confident about the state of the world – there can be no further information gain and epistemic value will be the same for all policies.

When there are no preferences, the most likely policies maximise uncertainty or expected information over outcomes (i.e., keep options open), in accord with the maximum entropy principle (Jaynes, 1957); while minimising the entropy of outcomes, given the state. Heuristically, this means agents will try to avoid uninformative (low entropy) *outcomes* (e.g., closing one’s eyes), while avoiding *states* that produce ambiguous (high entropy) outcomes (e.g., a noisy restaurant) (Schwartenbeck et al., 2013). This resolution of uncertainty is closely related to satisfying artificial curiosity (Schmidhuber, 1991, Still and Precup, 2012) and speaks to the value of information (Howard, 1966). It is also referred to as intrinsic value: see (Barto et al., 2004) for discussion of intrinsically motivated learning. Epistemic value can be regarded as the drive for novelty seeking behaviour (Wittmann et al., 2008, Krebs et al., 2009, Schwartenbeck et al., 2013), in which we anticipate the resolution of uncertainty (e.g., opening a birthday present). See also (Barto et al., 2013).

The expected complexity or cost is exactly the same quantity minimised in risk sensitive or KL control (Klyubin et al., 2005, van den Broek et al., 2010), and underpins related (free energy) formulations of bounded rationality based on complexity costs (Braun et al., 2011, Ortega and Braun, 2013). In other words, minimising expected complexity renders behaviour risk-sensitive, while maximising expected accuracy renders behaviour ambiguity-sensitive.

Although the above expressions appear complicated, expected free energy can be expressed in a compact and simple form in terms of the generative model:

 (6)

The two terms in the expression for expected free energy represent risk and ambiguity sensitive contributions respectively, where utility is a vector of preferences over outcomes. The decomposition of expected free energy in terms of expected cost and ambiguity lends a formal meaning to risk and ambiguity: risk is the relative entropy or uncertainty about outcomes, in relation to preferences, while ambiguity is the uncertainty about outcomes in relation to the state of the world. This is largely consistent with the use of risk and ambiguity in economics (Kahneman and Tversky, 1979, Zak, 2004, Knutson and Bossaerts, 2007, Preuschoff et al., 2008), where ambiguity reflects uncertainty about the context (e.g., which lottery is currently in play).

In summary, the above formalism suggests that expected free energy can be carved in two complementary ways: it can be decomposed into a mixture of epistemic and extrinsic value, promoting explorative, novelty-seeking and exploitative, reward-seeking behaviour respectively. Equivalently, minimising expected free energy can be formulated as minimising a mixture of expected cost or risk and ambiguity. This completes our description of free energy. We now turn to belief updating that is based on minimising free energy under the generative model described above.

### 2.4 Belief updating

Belief updating mediates inference and learning, where inference means optimising expectations about hidden states (policies and precision), while learning refers to optimising model parameters. This optimisation entails finding the sufficient statistics of posterior beliefs that minimise variational free energy. These solutions are (see Appendix A):(7)
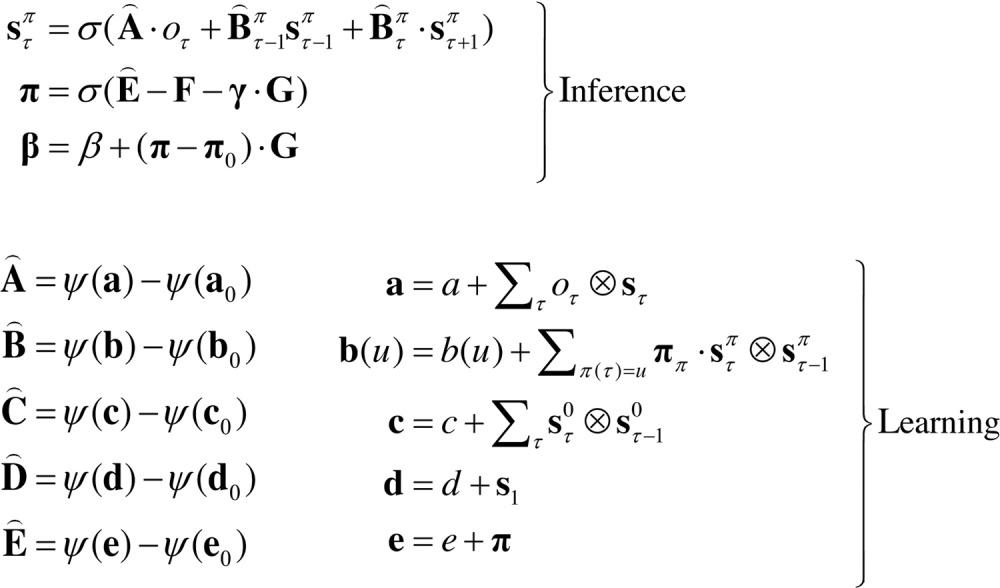


For notational simplicity, we have used: ${\overset{⌢}{B}}_{\tau }^{\pi }=\overset{⌢}{B}(\pi (\tau ))$, ${\overset{⌢}{B}}_{\tau }^{0}=\overset{⌢}{C}$, $\overset{⌢}{D}={\overset{⌢}{B}}_{0}^{\pi }s_{0}^{\pi }$, γ=1/β and $\pi_{0}=\sigma (\overset{⌢}{E}-\gamma \cdot G)$.

Usually, in variational Bayes, one would iterate the above self-consistent equations until convergence. However, we can also obtain the solution in a robust and biologically more plausible fashion by using a gradient descent on free energy (see Friston et al., under review): Solving these equations produces posterior expectations that minimise free energy to provide Bayesian estimates of hidden variables. This means that expectations change over several timescales: a fast timescale that updates posterior beliefs about hidden states after each observation (to minimise free energy over peristimulus time) and a slower timescale that updates posterior beliefs as new observations are sampled (to mediate evidence accumulation over observations); see also (Penny et al., 2013). Finally, at the end of each sequence of observations (i.e., trial of observation epochs) the expected (concentration) parameters are updated to mediate learning over trials. These updates are remarkably simple and have intuitive (neurobiological) interpretations:

Updating hidden states correspond to *state estimation*, under each policy. Because each expectation is informed by expectations about past and future states, this scheme has the form of a Bayesian smoother that combines (empirical) prior expectations about hidden states with the likelihood of the current observation. Having said this, the scheme does not use conventional forward and backward sweeps, because all future and past states are encoded explicitly. In other words, representations always refer to the same hidden state at the same time in relation to the start of the trial – not in relation to the current time. This may seem counterintuitive but this form of spatiotemporal (place and time) encoding finesses belief updating considerably and has a degree of plausibility in relation to empirical findings, as discussed elsewhere (Friston and Buzsaki, 2016).

The policy updates are just a softmax function of their log probability, which has three components: a prior based on previous experience, the (posterior) free energy based on past outcomes and the expected (prior) free energy based on preferences about future outcomes. Note that prior beliefs about policies in the generative model are supplemented or informed by the (posterior) free energy based on outcomes. Because habits are just another policy, the arbitration among habits and (sequential) policies rests on their posterior probability, which is closely related to the proposals in (Daw et al., 2005, Lee et al., 2014) but introduces a risk and ambiguity trade-off in policy selection (FitzGerald et al., 2014). Policy selection also entails the optimisation of expected uncertainty or precision. This is expressed above in terms of the temperature (inverse precision) of posterior beliefs about precision: β=1/γ. One can see that temperature increases with expected free energy. In other words, policies that, on average, have a high expected free energy will influence posterior beliefs about policies with less precision.

Interestingly, the updates to temperature (and implicitly precision) are determined by the difference between the expected free energy under posterior beliefs about policies and the expected free energy under prior beliefs. This endorses the notion of *reward prediction errors* as an explanation for dopamine responses; in the sense that if posterior beliefs based upon current observations reduce the expected free energy, relative to prior beliefs, then precision will increase (FitzGerald et al., 2015a, FitzGerald et al., 2015b). This can be related to dopamine discharges that have been interpreted in terms of changes in expected reward (Schultz and Dickinson, 2000, Fiorillo et al., 2003). The role of the neuromodulator dopamine in encoding precision is also consistent with its multiplicative effect in the second update – to nuance the selection among competing policies (Fiorillo et al., 2003, Frank et al., 2007, Humphries et al., 2009, Humphries et al., 2012, Solway and Botvinick, 2012, Mannella and Baldassarre, 2015). We will return to this later.

Finally, the updates for the parameters bear a marked resemblance to classical Hebbian plasticity (Abbott and Nelson, 2000). The transition or connectivity updates comprise two terms: an associative term that is a digamma function of the accumulated coincidence of past (postsynaptic) and current (presynaptic) states (or observations under hidden causes) and a decay term that reduces each connection as the total afferent connectivity increases. The associative and decay terms are strictly increasing but saturating functions of the concentration parameters. Note that the updates for the (connectivity) parameters accumulate coincidences over time because, unlike hidden states, parameters are time invariant. Furthermore, the parameters encoding state transitions have associative terms that are modulated by policy expectations. In addition to the learning of contingencies through the parameters of the transition matrices, the vectors encoding beliefs about the initial state and selected policy accumulate evidence by simply counting the number of times they occur. In other words, if a particular state or policy is encountered frequently, it will come to dominate posterior expectations. This mediates *context learning* (in terms of the initial state) and *habit learning* (in terms of policy selection). In practice, the learning updates are performed at the end of each trial or sequence of observations. This ensures that learning benefits from inferred (postdicted) states, after ambiguity has been resolved through epistemic behaviour. For example, the agent can learn about the initial state, even if the initial cues were completely ambiguous.

### 2.5 Summary

**Fig. 2:**
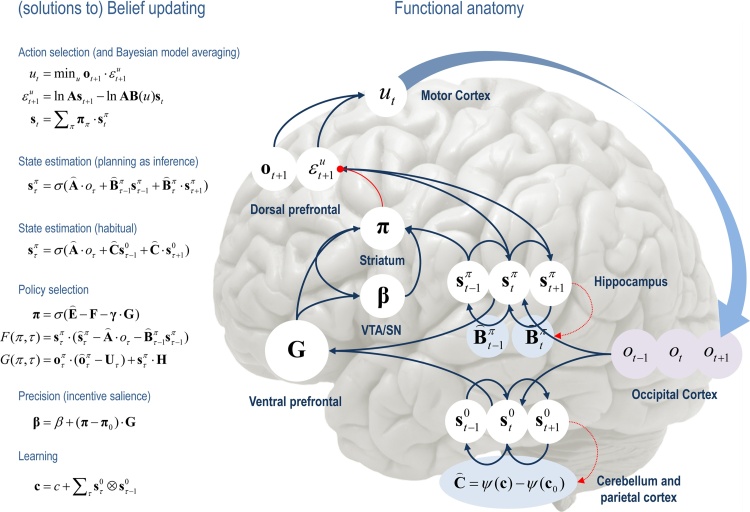
Overview of belief updates for discrete Markovian models: the left panel lists the solutions in the main text, associating various updates with action, perception, policy selection, precision and learning. The right panel assigns the variables (sufficient statistics or expectations) to various brain areas to illustrate a rough functional anatomy – implied by the form of the belief updates. Observed outcomes are signed to visual representations in the occipital cortex. State estimation has been associated with the hippocampal formation and cerebellum (or parietal cortex and dorsal striatum) for planning and habits respectively (Everitt and Robbins, 2013). The evaluation of policies, in terms of their (expected) free energy, has been placed in the ventral prefrontal cortex. Expectations about policies per se and the precision of these beliefs have been assigned to striatal and ventral tegmental areas to indicate a putative role for dopamine in encoding precision. Finally, beliefs about policies are used to create Bayesian model averages of future states (over policies) – that are fulfilled by action. The blue arrows denote message passing, while the solid red line indicates a modulatory weighting that implements Bayesian model averaging. The broken red lines indicate the updates for parameters or connectivity (in blue circles) that depend on expectations about hidden states (e.g., associative plasticity in the cerebellum). Please see the appendix for an explanation of the equations and variables. The large blue arrow completes the action perception cycle, rendering outcomes dependent upon action. (For interpretation of the references to colour in this figure legend, the reader is referred to the web version of this article.)

By assuming a generic (Markovian) form for the generative model, it is fairly easy to derive Bayesian updates that clarify the relationships between perception, policy selection, precision and action – and how these quantities shape beliefs about hidden states of the world and subsequent behaviour. In brief, the agent first infers the hidden states under each model or policy that it entertains. It then evaluates the evidence for each policy based upon prior beliefs or preferences about future outcomes. Having optimised the precision or confidence in beliefs about policies, they are used to form a Bayesian model average of the next outcome, which is realised through action. The anatomy of the implicit message passing is not inconsistent with functional anatomy in the brain: see (Friston et al., 2014) and Fig. 1, Fig. 2. Fig. 2 reproduces the (solutions to) belief updating and assigns them to plausible brain structures. This functional anatomy rests on reciprocal message passing among expected policies (e.g., in the striatum) and expected precision (e.g., in the substantia nigra). Expectations about policies depend upon expected outcomes and states of the world (e.g., in the prefrontal cortex (Mushiake et al., 2006) and hippocampus (Pezzulo et al., 2014, Pezzulo and Cisek, 2016, Stoianov et al., 2016)). Crucially, this scheme entails reciprocal interactions between the prefrontal cortex and basal ganglia (Botvinick and An, 2008, Pennartz et al., 2011, Verschure et al., 2014); in particular, selection of expected (motor) outcomes by the basal ganglia (Mannella and Baldassarre, 2015). In the next section, we consider the formal relationships between active inference and conventional schemes based upon value functions.

## 3 Relationship to Bellman formulations

Hitherto, we have assumed that habits are based upon learned state transitions. However, it is possible that these transitions could be evaluated directly, under the assumption that an optimal (state-action) policy will be adopted in the future. Dynamic programming or backwards induction is the standard approach to optimising state-action policies under this assumption (Bellman, 1952, Howard, 1960). We can express dynamic programming using the above notation as follows:(8)
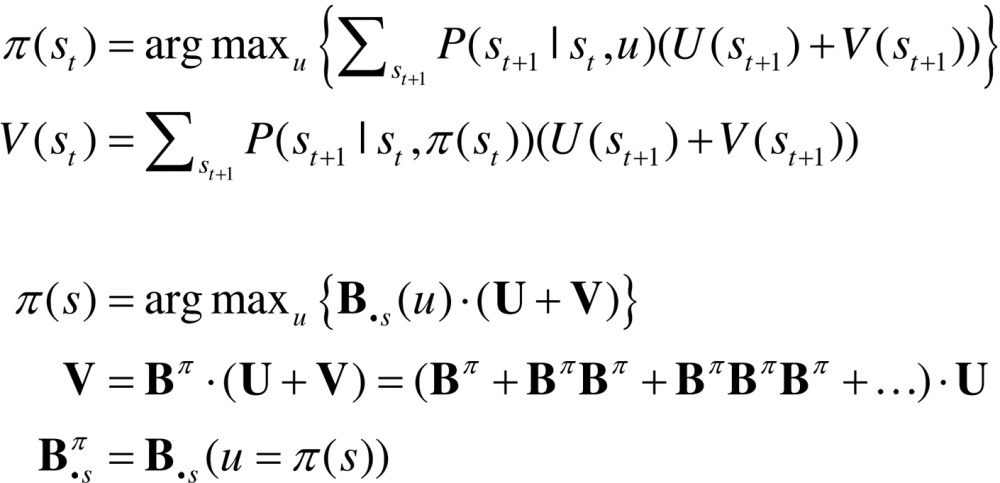


The first pair of equations represents the two steps of dynamic programming. The second set of equations expresses the optimal policy in terms of our generative model, where $B_{s}$ denotes the column of the matrix encoding the transitions from state *s*. In brief, the optimal policy returns the action that maximises utility U(s)∈U plus a value-function of states V(s)∈V. The value-function is then evaluated under the optimal policy, until convergence. The value-function represents the expected utility (cf., prior preference) integrated over future states. The close relationship between dynamic programming and backwards induction is highlighted by the final expression for value, which is effectively the utility over states propagated backwards in time by the optimal (habitual) transition matrix.

Dynamic programming supposes that there is an optimal action that can be taken from every state, irrespective of the context or time of action. This is, of course, the same assumption implicit in habit learning − and we might expect to see a correspondence between the state transitions encoded by **C** = **B**^0^ and $B^{\pi }$ (we will return to this in the last section). However, this correspondence will only arise when the (Bellman) assumptions of dynamic programming or backwards induction hold; i.e., when states are observed unambiguously, such that o=s and U(o)=U(s)∈U. In these cases, one can also use variational belief updating to identify the best action from any state. This is the action associated with the policy that minimises expected free energy, starting from any state:(9)
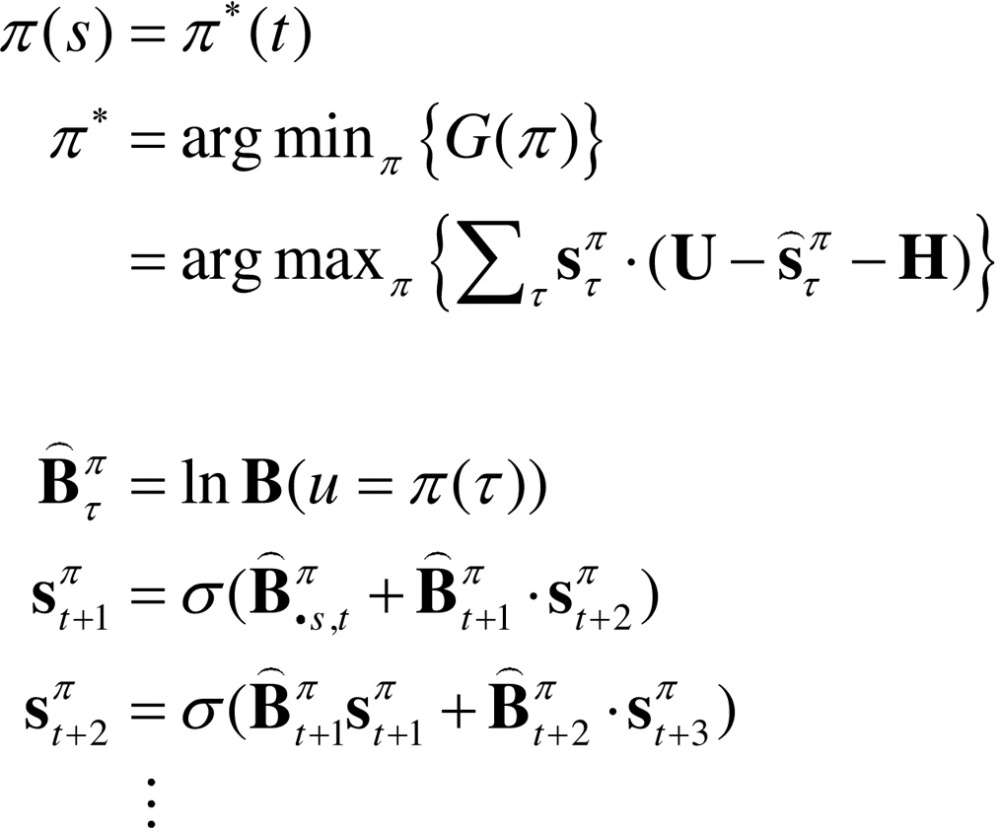


This effectively composes a state-action policy by picking the action under the best policy from each state (assuming the current state is known). The key point here is that dynamic programming is a special case of this variational scheme. One can see this by substituting the expression for value above into the first step of dynamic programming. This is known as direct policy iteration (Williams, 1992, Baxter et al., 2001). The ensuing policy iteration scheme can now be expressed, not in terms of value, but in terms of future states.(10)
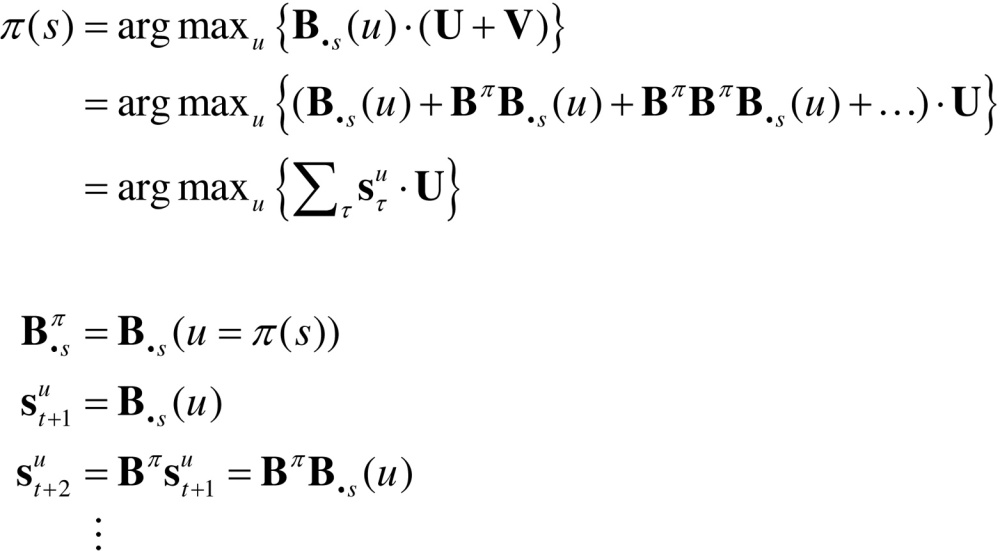


This is formally equivalent to the variational state-action policy with two differences. First, the policy iteration scheme simply maximises expected utility, as opposed to expected free energy. This means the risk and ambiguity terms disappear and free energy reduces to expected utility. The second difference pertains to the recursive iteration of future states: active inference uses variational updates to implement Bayesian smoothing, whereas the backward induction scheme imputes future states by recursive application of the optimal transition matrix.

One might question the relative merits of iteratively evaluating the value-function of states (Eq. (8)), as opposed to the states per se (Eq, (10)). Clearly, if one wants to deal with risk and ambiguity, then an evaluation of the states (and their entropy) is necessary. In other words, if one wants to augment conventional utility functions with risk and ambiguity terms, it becomes necessary to evaluate beliefs about future states (as in Eq. (10)). This has a profound implication for schemes (such as dynamic programming, backwards induction and reinforcement learning) based on value functions. These schemes are, in essence, belief-free because the construction of value functions precludes a contribution from beliefs about the future (unless one uses a belief MDP). This is a key difference between (belief-based) active inference and (belief-free) schemes based upon the Bellman assumptions. In summary, belief-free schemes are limited to situations in which there is no ambiguity about hidden states (which are difficult to conceive in most interesting or real-world settings). We will see an example of this limitation in the next section. This completes our theoretical treatment of active inference and learning. In the last section, we use simulations to revisit some key concepts above.

## 4 Simulations of foraging

This section considers inference and learning using simulations of foraging in a T-maze. This T-maze contains primary rewards (such as food) and cues that are not rewarding per se but disclose the location of rewards. The basic principles of this problem can be applied to any number of scenarios (e.g., saccadic eye movements to visual targets). This is the same setup used in (Friston et al., 2015) and is as simple as possible, while illustrating some key behaviours. Crucially, this example can also be interpreted in terms of responses elicited in reinforcement learning paradigms by *unconditioned* (US) and *conditioned* (CS) stimuli. Strictly speaking, our paradigm is instrumental and the cue is a *discriminative stimulus*; however, we will retain the Pavlovian nomenclature, when relating precision updates to dopaminergic discharges.

### 4.1 The setup

An agent (e.g., a rat) starts in the center of a T-maze, where either the right or left arms are baited with a reward (US). The lower arm contains a discriminative cue (CS) that tells the animal whether the reward is in the upper right or left arm. Crucially, the agent can only make two moves. Furthermore, the agent cannot leave the baited arms after they are entered. This means that the optimal behaviour is to first go to the lower arm to find where the reward is located and then retrieve the reward at the cued location.

In terms of a Markov decision process, there are four control states that correspond to visiting, or sampling, the four locations (the center and three arms). For simplicity, we assume that each action takes the agent to the associated location (as opposed to moving in a particular direction from the current location). This is analogous to place-based navigation strategies thought to be mediated by the hippocampus (Moser et al., 2008). There are eight hidden states: four locations times, two contexts (right and left reward) and seven possible outcomes. The outcomes correspond to being in the center of the maze plus the (two) outcomes at each of the (three) arms that are determined by the context (the right or left arm is more rewarding).

**Fig. 3:**
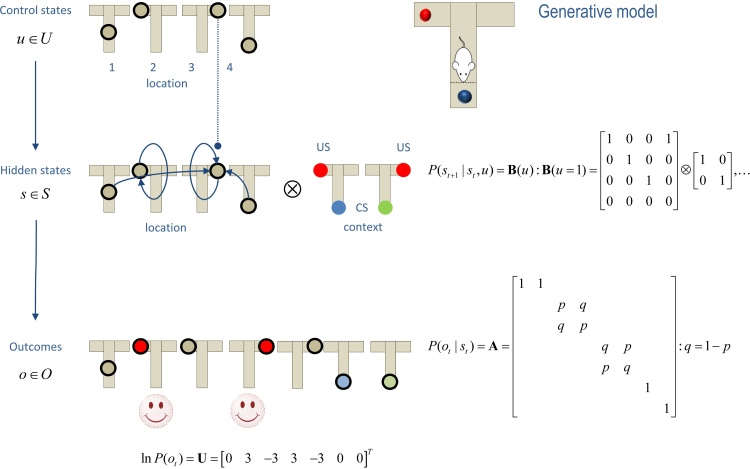
The generative model used to simulate foraging in a three-arm maze (insert on the upper right). This model contains four control states that encode movement to one of four locations (three arms and a central location). These control the transition probabilities among hidden states that have a tensor product form with two factors: the first is place (one of four locations), while the second is one of two contexts. These correspond to the location of rewarding (red) outcomes and the associated cues (blue or green circles). Each of the eight hidden states generates an observable outcome, where the first two hidden states generate the same outcome that just tells the agent that it is at the center. Some selected transitions are shown as arrows, indicating that control states attract the agent to different locations, where outcomes are sampled. The equations define the generative model in terms of its parameters (**A**,**B**), which encode mappings from hidden states to outcomes and state transitions respectively. The lower vector corresponds to prior preferences; namely, the agent expects to find a reward. Here, ⊗ denotes a Kronecker tensor product. (For interpretation of the references to colour in this figure legend, the reader is referred to the web version of this article.)

Having specified the state-space, it is now necessary to specify the (**A**,**B**) matrices encoding contingencies. These are shown in Fig. 3, where the **A** matrix maps from hidden states to outcomes, delivering an ambiguous cue at the center (first) location and a definitive cue at the lower (fourth) location. The remaining locations provide a reward (or not) with probability p=98% depending upon the context. The B(u) matrices encode action-specific transitions, with the exception of the baited (second and third) locations, which are (absorbing) hidden states that the agent cannot leave.

One could consider learning contingencies by updating the prior concentration parameters (a,b) of the transition matrices but we will assume the agent knows (i.e., has very precise beliefs about) the contingencies. This corresponds to making the prior concentration parameters very large. Conversely, we will use small values of (c,d) to enable habit and context learning respectively. The parameters encoding prior expectations about policies (e) will be used to preclude (this section) or permit (next section) the selection of habitual policies. Preferences in the vector $U_{\tau }=\ln P(o_{\tau })$ encode the utility of outcomes. Here, the utilities of a rewarding and unrewarding outcome were 3 and −3 respectively (and zero otherwise). This means, the agent expects to be rewarded exp(3)≈20 times more than experiencing a neutral outcome. Note that utility is always relative and has a quantitative meaning in terms of preferred states. This is important because it endows utility with the same measure as information; namely, nats (i.e., units of information or entropy based on natural logarithms). This highlights the close connection between value and information.

Having specified the state-space and contingencies, one can solve the belief updating equations (Eq. (7)) to simulate behaviour. The (concentration) parameters of the habits were initialised to the sum of all transition probabilities: $c=\sum_{u}B(u)$. Prior beliefs about the initial state were initialised to d=8 for the central location for each context and zero otherwise. Finally, prior beliefs about policies were initialised to e=4 with the exception of the habit, where e=0. These concentration parameters can be regarded as the number of times each state, transition or policy has been encountered in previous trials.

**Fig. 4:**
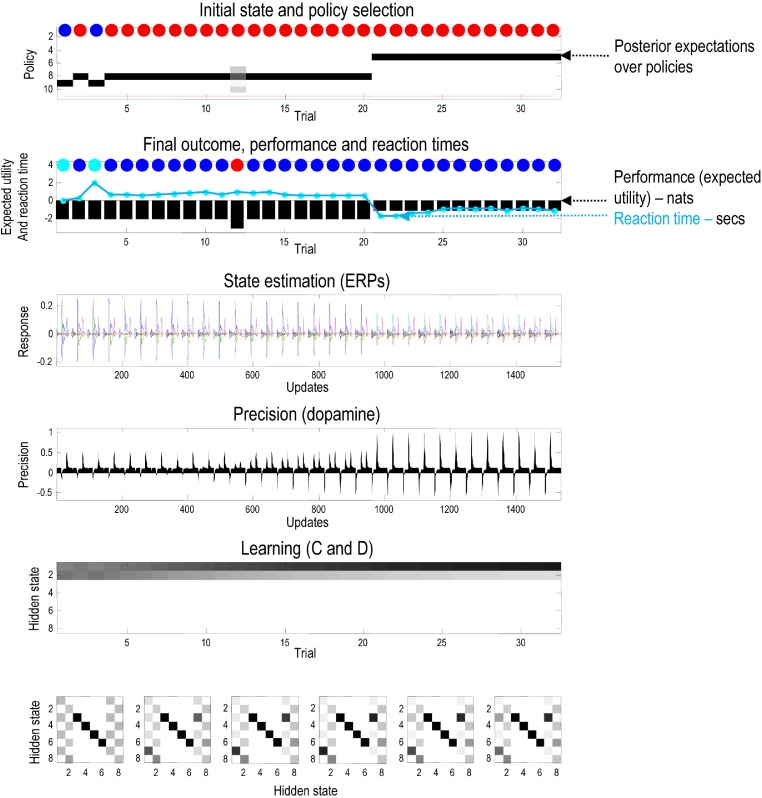
Simulated responses over 32 trials: this figure reports the behavioural and (simulated) physiological responses during successive trials. The first panel shows, for each trial, the initial state (as blue and red circles indicating the context) and the selected policy (in image format) over the 11 policies considered. The policies are selected in the first two trials correspond to epistemic policies (#8 and #9), which involve examining the cue in the lower arm and then going to the left or right arm to secure the reward (depending on the context). After the agent becomes sufficiently confident that the context does not change (after trial 21) it indulges in pragmatic behaviour, accessing the reward directly. The red line shows the posterior ability of selecting the habit, which is was set to zero in these simulations. The second panel reports the final outcomes (encoded by coloured circles: cyan and blue for rewarding outcomes in the left and right arms) and performance measures in terms of preferred outcomes, summed over time (black bars) and reaction times (cyan dots). The third panel shows a succession of simulated event related potentials following each outcome. These are taken to be the rate of change of neuronal activity, encoding the expected probability of hidden states. The fourth panel shows phasic fluctuations in posterior precision that can be interpreted in terms of dopamine responses. The final two panels show context and habit learning, expressed in terms of (**C**,**D**): the penultimate panel shows the accumulated posterior beliefs about the initial state, while the lower panels show the posterior expectations of habitual state transitions. Here, each panel shows the expected transitions among the eight hidden states (see Fig. 3), where each column encodes the probability of moving from one state to another. Please see main text for a detailed description of these responses. (For interpretation of the references to colour in this figure legend, the reader is referred to the web version of this article.)

Fig. 4 summarises the (simulated) behavioural and physiological responses over 32 successive trials using a format that will be adopted in subsequent figures. Each trial comprises two actions following an initial observation. The top panel shows the initial states on each trial (as coloured circles) and subsequent policy selection (in image format) over the 11 policies considered. The first 10 (allowable) policies correspond to staying at the center and then moving to each of the four locations, moving to the left or right arm and staying there, or moving to the lower arm and then moving to each of the four locations. The 11th policy corresponds to a habit (i.e., state-action policy). The red line shows the posterior probability of selecting the habit, which is effectively zero in these simulations because we set its prior (concentration parameter) to zero. The second panel reports the final outcomes (encoded by coloured circles) and performance. Performance is reported in terms of preferred outcomes, summed over time (black bars) and reaction times (cyan dots). Note that because preferences are log probabilities they are always negative – and the best outcome is zero.^2^ The reaction times here are based upon the processing time in the simulations (using the Matlab *tic-toc* facility) and are shown after normalisation to a mean of zero and standard deviation of one.

In this example, the first couple of trials alternate between the two contexts with rewards on the right and left. After this, the context (indicated by the cue) remained unchanged. For the first 20 trials, the agent selects epistemic policies, first going to the lower arm and then proceeding to the reward location (i.e., left for *policy #8* and right for *policy #*9). After this, the agent becomes increasingly confident about the context and starts to visit the reward location directly. The differences in performance between these (epistemic and pragmatic) behaviours are revealed in the second panel as a decrease in reaction time and an increase in the average utility. This increase follows because the average is over trials and the agent spends two trials enjoying its preferred outcome, when seeking reward directly – as opposed to one trial when behaving epistemically. Note that on trial 12, the agent received an unexpected (null) outcome that induces a degree of posterior uncertainty about which policy it was pursuing. This is seen as a non-trivial posterior probability for three policies: the correct (context-sensitive) epistemic policy and the best alternatives that involve staying in the lower arm or returning to the center.

The third panel shows a succession of simulated event related potentials following each outcome. These are the rate of change of neuronal activity, encoding the expected probability of hidden states. The fourth panel shows phasic fluctuations in posterior precision that can be interpreted in terms of dopamine responses. Here, the phasic component of simulated dopamine responses corresponds to the rate of change of precision (multiplied by eight) and the tonic component to the precision per se (divided by eight). The phasic part is the precision prediction error (cf., reward prediction error: see Eq. (8)). These simulated responses reveal a phasic response to the cue (CS) during epistemic trials that emerges with context learning over repeated trials. This reflects an implicit transfer of dopamine responses from the US to the CS. When the reward (US) is accessed directly there is a profound increase in the phasic response, relative to the response elicited after it has been predicted by the CS.

The final two panels show context and habit learning: the penultimate panel shows the accumulated posterior expectations about the initial state **D**, while the lower panels show the posterior expectations of habitual state transitions, **C**. The implicit learning reflects an accumulation of evidence that the reward will be found in the same location. In other words, initially ambiguous priors over the first two hidden states come to reflect the agent’s experience that it always starts in the first hidden state. It is this context learning that underlies the pragmatic behaviour in later trials. We talk about context learning (as opposed to inference) because, strictly speaking, Bayesian updates to model parameters (between trials) are referred to as learning, while updates to hidden states (within trial) correspond to inference.

Finally, the expected state transitions under a habitual policy show the emergence of an epistemic policy, in which the agent always goes to the lower (fourth) location from the central (first) location, irrespective of context. It then locates the appropriate (second or third) locations. It is more confident about vicarious transitions to the second location, because these predominate in its recent experience. The next section considers learning in more detail, looking first at context learning and then habit learning.

## 5 Simulations of learning

This section illustrates the distinction between *context* and *habit* learning. In the previous section, context learning enabled more informed and confident (pragmatic) behaviour as the agent became familiar with its environment. In this section, we consider how the same context learning can lead to perseveration and thereby influence reversal learning, when contingencies change. Following this, we turn to habit learning and simulate some cardinal aspects of devaluation. Finally, we turn to epistemic habits and close by comparing an acquired with and without ambiguous outcomes. This serves to highlight the difference between belief-based and belief-free schemes – and illustrates the convergence of active inference and belief-free schemes, when the world is fully observed.

### 5.1 Context and reversal learning

**Fig. 5:**
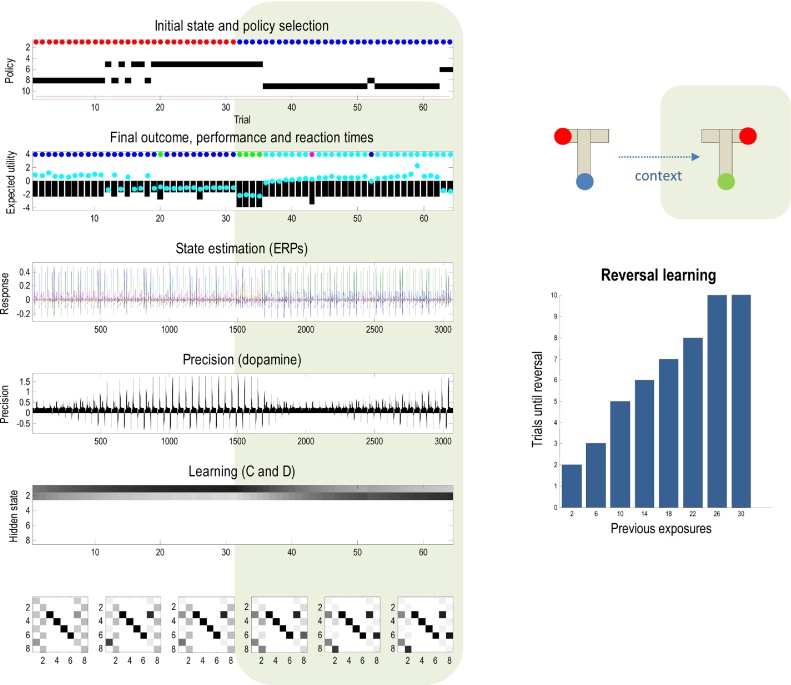
Reversal learning: this figure uses the format of Fig. 4 to illustrate behavioural and physiological responses induced by reversal learning. In this example, 64 trials were simulated with a switch in context from one (consistent) reward location to another. The upper panel shows that after about 16 trials the agent is sufficiently confident about the context to go straight to the rewarding location; thereby switching from an epistemic to a pragmatic policy. After 32 trials the context changes but the (pragmatic) policy persists; leading to 4 trials in which the agent goes to the wrong location. After this, it reverts to an epistemic policy and, after a period of context learning, adopts a new pragmatic policy. Behavioural perseveration of this sort is mediated purely by prior beliefs about context that accumulate over trials. This is illustrated in the right panel, which shows the number of perseverations after reversal, as a function of the number of preceding (consistent) trials.

Fig. 5 uses the format of Fig. 4 to illustrate behavioural and physiological responses induced by reversal learning. In this example, 64 trials were simulated with a switch in context to a (consistent) reward location from the left to the right arm after 32 trials. The upper panel shows that after about 16 trials the agent is sufficiently confident about the context to go straight to the rewarding location; thereby switching from an epistemic to a pragmatic policy. Prior to this switch, phasic dopamine responses to the reward (US) progressively diminish and are transferred to the discriminative cue (CS) (Fiorillo et al., 2003). After adopting a pragmatic policy, dopamine responses to the US disappear because they are completely predictable and afford no further increase in precision.

Crucially, after 32 trials the context changes but the (pragmatic) policy persists, leading to 4 trials in which the agent goes to the wrong location. After this, it reverts to an epistemic policy and, after a period of context learning, adopts a new pragmatic policy. Behavioural perseveration of this sort is mediated purely by prior beliefs about context that accumulate over trials. Here, this is reflected in the prior belief about the hidden states encountered at the beginning of each new trial (shown as a function of trials in the fifth panel). This context learning is illustrated in the right panel, which shows the number of perseverative trials before reversal, as a function of previous exposures to the original context.

Note that this form of reversal learning reflects changes in prior expectations about the hidden states generating the first outcome. This should be contrasted with learning a reversal of contingencies encoded by the state transition parameters, or parameters mapping from states to outcomes. Learning these parameters would also produce reversal learning and a number of other phenomena in psychology; such as effect of partial reinforcement (Delamater and Westbrook, 2014). However, in this paper, we focus on context and habit learning; as opposed to *contingency* learning. The above demonstration of reversal learning proceeded in the absence of habits. In the remaining simulations, we enabled habit learning by allowing its (concentration) parameter to accumulate over trials.

### 5.2 Habit formation and devaluation

**Fig. 6:**
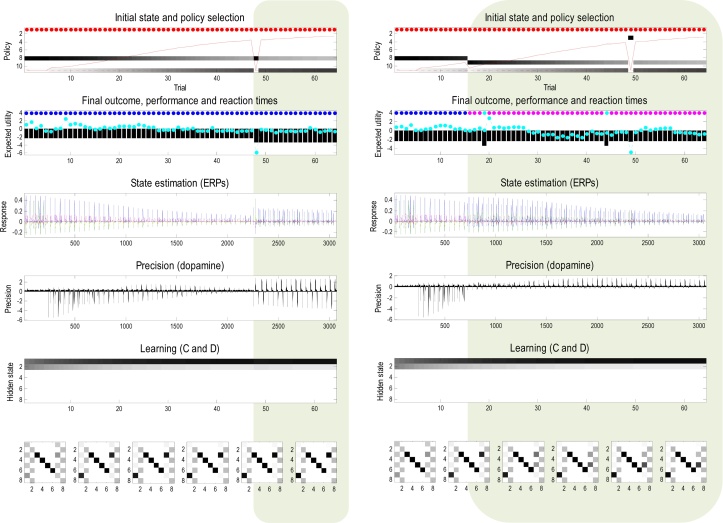
Habit formation and devaluation: this figure uses the same format as the previous figure to illustrate habit formation and the effects of devaluation. The left panels show habit learning over 64 trials in which the context was held constant. The posterior probability of the habitual policy is shown in the upper panel (solid red line), where the habit is underwritten by the state transitions shown in the lower panels. The simulation shows that as the habitual transitions are learnt, the posterior probability of the habit increases until it is executed routinely. After the habit had been acquired, we devalued the reward by switching the prior preferences such that the neutral outcome became the preferred outcome (denoted by the green shaded areas). Despite this preference reversal, the habit persists. The right panels report the same simulation when the reward was devalued after 16 trials, before the habit was fully acquired. In this instance, the agent switches immediately to the new preference and subsequently acquires a habit that is consistent with its preferences (compare the transition probabilities in the lower panels). (For interpretation of the references to colour in this figure legend, the reader is referred to the web version of this article.)

Fig. 6 uses the same format as the previous figure to illustrate habit formation and the effects of devaluation. Devaluation provides a critical test for dissociable (goal-directed or contingency and habitual or incentive) learning mechanisms in psychology (Balleine and Dickinson, 1998, Yin and Knowlton, 2006). The left-hand panels show habit learning over 64 trials in which the context was held constant. The posterior probability of the habitual policy is shown in the upper panel (solid red line), where the habit is underwritten by the state transitions in the lower panels. This simulation shows that as habitual transitions are learnt, the posterior probability of the habit increases until it is executed routinely. In this case, the acquired habit corresponds to an epistemic policy (*policy #8*), and after the habit has been acquired, there is no opportunity for pragmatic policies. This means that although the behaviour is efficient in terms of reaction times, the habit has precluded exploitative behaviour (Dayan et al., 2006). The reason why this habit has epistemic components is because it was learned under prior beliefs that both contexts were equally likely; conversely, a habit acquired under a different prior could be pragmatic.

One might ask why a habit is selected over a sequential policy that predicts the same behaviour. The habit is selected because it provides a better explanation for observed outcomes. This is because the joint distribution over successive states is encoded by the concentration parameters c⊂η (see Eq. (6)). Technically, this means that habits have less complexity and free energy path integrals. One can see this anecdotally in the transition matrices on the lower left of Fig. 6: if we were in the seventh state after the first move, we can be almost certain we started in the first state. However, under the model of transitions provided by the best sequential policy (*policy* #8), the empirical prior afforded by knowing we were in the seventh state is less definitive (we could have moved from the first state or we could have already been in the seventh).

During the acquisition of the habit, the reaction times decrease with maintained performance and systematic changes in phasic dopamine responses (fourth panel). An important correlate of habit learning is the attenuation of electrophysiological responses (e.g., in the hippocampus). This reflects the fact that the equivalent belief updates for the habit (e.g., in the cerebellum, parietal cortex and dorsolateral striatum (Everitt and Robbins, 2013)), have been deliberately omitted from the graphics. This effective transfer of sequential processing (from hippocampus to cerebellar cortex) may provide a simple explanation for the putative transfer in real brains during memory consolidation; for example, during sleep (Buzsaki, 1998, Kesner, 2000, Pezzulo et al., 2014).

Crucially, after the habit was acquired the reward was devalued by switching the prior preferences (at trial 48), such that the neutral outcome became the preferred outcome (denoted by the green shaded areas). Despite this switch, the habit persists and, indeed, reinforces itself with repeated executions. The right panels report exactly the same simulation when the rewards were devalued after 16 trials, before the habit was fully acquired. In this instance, the agent switches its behaviour immediately (before sampling the devalued outcome) and subsequently acquires a habit that is consistent with its preferences (compare the transition probabilities in the lower panels). In other words, prior to habit formation, goal directed behaviour is sensitive to devaluation – a sensitivity that is lost under habitual control. These simulations demonstrate the resistance of habitual policies to devaluation resulting in suboptimal performance (but faster reaction times: see second panel). See Dayan et al. (2006) for a discussion of how habits can confound learning in this way.

### 5.3 Epistemic habit acquisition under ambiguity

**Fig. 7:**
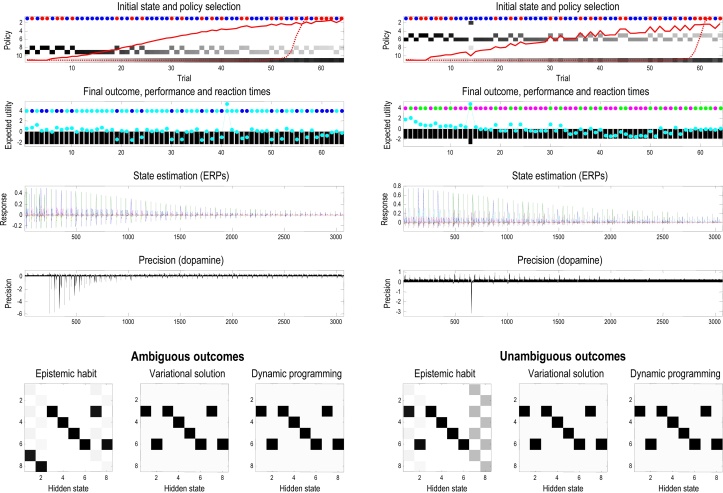
Epistemic habit acquisition under ambiguity: this figure uses the same format as Fig. 6 to illustrate the acquisition of epistemic habits under ambiguous (left panels) and unambiguous (right panels) outcomes. The left panels show the rapid acquisition of an epistemic habit after about 16 trials of epistemic cue-seeking, when the context switches randomly from one trial to the next. The lower panels show the state transitions under the habitual policy; properly enforcing a visit to the cue location followed by appropriate reward seeking. This policy should be contrasted with the so-called optimal policy provided by dynamic programming (and the equivalent variational estimate) in the lower panels. The optimal (epistemic) state-action policy is fundamentally different from the optimal (pragmatic) habit under dynamic programming. This distinction can be dissolved by making the outcomes unambiguous. The right panels report the results of an identical simulation, where outcomes observed from the starting location specify the context unambiguously.

Fig. 7 illustrates the acquisition of epistemic habits under ambiguous (left panels) and unambiguous (right panels) outcome contingencies. In these simulations, the context switches randomly from one trial to the next. The left panels show the rapid acquisition of an epistemic habit after about 16 trials of epistemic cue-seeking. As the agent observes its own habitual behaviour, the prior probability of the habit increases (dotted red line in the upper panel). This prior probability is based upon the policy concentration parameters, e⊂η. The lower panels show the state transitions under the habitual policy; properly enforcing a visit to the cue location followed by appropriate reward seeking.

This policy should be contrasted with the so-called optimal policy provided by dynamic programming (and the equivalent variational estimate) in the lower panels: these are the solutions to Eqs. (9) and (10). Clearly, the ‘optimal’ policy is to go straight to the rewarding location in each context (or hidden state); however, this is no use when outcomes are ambiguous and the agent does not know which context it is in. This means the optimal (epistemic) state-action policy under active inference (left panel) is fundamentally different from the optimal (pragmatic) habit under dynamic programming (right panel). This distinction can be dissolved by making the outcomes unambiguous. The right panels report the results of an identical simulation with one important difference – the outcomes observed from the starting location unambiguously specify the context. In this instance, all state-action policies are formally identical (although transitions from the cue location are not evaluated under active inference, because they are never encountered).

### 5.4 Summary

In summary, these simulations suggest that agents should acquire epistemic habits – and can only do so through belief-based learning. There is nothing remarkable about epistemic habits; they are entirely consistent with the classical conception of habits – in the animal learning literature – as chains of stimulus-response associations. The key aspect here is that they can be acquired (autodidactically) via observing epistemic goal-directed behaviour.

## 6 Conclusion

We have described an active inference scheme for discrete state-space models of choice behaviour that is suitable for modelling a variety of paradigms and phenomena. Although goal-directed and habitual policies are usually considered in terms of *model-based* and *model-free* schemes, we find the more important distinction is between *belief-free* versus *belief-based* schemes; namely, whether the current state is sufficient to specify an action or whether it is necessary to consider beliefs about states (e.g., uncertainty). Furthermore, we show that conventional formulations (based on the Bellman optimality principle) apply only in the belief-free setting, when cues are unambiguous. Finally, we show how habits can emerge naturally from goal-directed behaviour.

To the extent that one accepts the variational (active inference) formulation of behaviour, there are interesting implications for the distinction between habitual and goal-directed behaviour. If we associate model-free learning with habit-learning, then model-free learning emerges from model-based behaviour. In other words, model-based planning engenders and contextualises model-free learning. In this sense, active inference suggests there can be no model-free scheme that is learned autonomously or divorced from goal-directed (model-based) behaviour. There are further implications for the role of value-functions and backwards induction in standard approaches to model-based planning. Crucially, variational formulations do not refer to value-functions of states, even when optimising habitual (state-action) policies. Put simply, learning in active inference corresponds to optimising the parameters of a generative model. In this instance, the parameters correspond to state transitions that lead to valuable (preferred) states. At no point do we need to learn an intermediary value-function from which these transitions are derived. In sum, the important distinction between goal-directed and habitual behaviour may not be the distinction between model-based and model-free but the distinction between selecting policies that are and are not sensitive to context or ambiguity; i.e. belief-based versus belief-free.

One might ask whether active inference makes any predictions about responses that have yet to be observed empirically. At the level of behavioural predictions, the answer is probably no. This follows from something called the *complete class theorem* (Brown, 1981), which states that for any observed behaviour and utility function there exists a prior that renders the behaviour Bayes optimal. Because active inference absorbs utility functions into prior preferences, this means there is always a set of prior preferences that renders any behaviour (approximately) Bayes optimal. At first glance, this may seem disappointing; however, turning the argument on its head, the complete class theorem means that we can always characterise behaviour in terms of prior preferences. This is important because it means one can computationally phenotype any behaviour and start to quantify – and understand – the prior beliefs that subjects bring to any paradigm. This is a tenet of computational psychiatry (Huys et al., 2011, Montague et al., 2012, Wang and Krystal, 2014), which motivates much of the work reported above.

At the level of the particular (neuronal) process theory described in this paper, there are many predictions about the neuronal correlates of perception, evaluation, policy selection and the encoding of uncertainty associated with dopaminergic discharges. For example, the key difference between expected free energy and value is the epistemic component or information gain. This means that a strong prediction (which to our knowledge has not yet been tested) is that a mildly aversive outcome that reduces uncertainty about the experimental or environmental context will elicit a positive phasic dopaminergic response.

## Disclosure statement

The authors have no disclosures or conflict of interest.

## Figures and Tables

**Table 1:** Glossary of expressions.

## Appendix A

*Belief updating*: variational updates are a self-consistent set of equalities that minimise variational free energy, which can be expressed as the (time-dependent) free energy under each policy plus the complexity incurred by posterior beliefs about (time-invariant) policies and parameters, where (ignoring constants and using η={a,b,c,d,e,β});

Free energy and its expectation are given by:

Here, ${\overset{⌢}{B}}_{\tau }^{\pi }=\overset{⌢}{B}(\pi (\tau ))$, ${\overset{⌢}{B}}_{\tau }^{0}=\overset{⌢}{C}$ and ${\overset{⌢}{B}}_{0}^{\pi }s_{0}^{\pi }=\overset{⌢}{D}$. B(d) is the beta function of the column vector **d** and the remaining variables are:

Using the standard result: $\partial_{d}B(d)=B(d)\overset{⌢}{D}$, we can differentiate the variational free energy with respect to the sufficient statistics (with a slight abuse of notation and using $\partial_{s}F≔\partial F(\pi ,\tau )/\partial s_{\pi }^{\tau }$):

Finally, the solutions to these equations give the variational updates in the main text (Eq. (7)).

## Footnotes

1 Here, we mean context insensitive in the sense of Thrailkill and Bouton (2015). In other words, context refers to outcome contingencies; not the paradigmatic context.

2 Utilities can only be specified to within an additive constant (the log normalisation constant) because of the sum to one constraint of probabilities. This means that although preferred outcomes were specified with utilities between −3 and +3, the actual utilities are negative.
